# Trends and Outcomes of Anticoagulation for Post‐Operative Atrial Fibrillation After Coronary Artery Bypass Graft

**DOI:** 10.1111/pace.70069

**Published:** 2025-10-23

**Authors:** Daniel S. Cheah, Kathryn Tsai, Krishna Prasad Kurpad, Yousra Khalid, Sanjay S. Mehta

**Affiliations:** ^1^ Carle Illinois College of Medicine Urbana Illinois USA; ^2^ Department of Cardiology Carle Foundation Hospital Urbana Illinois USA; ^3^ Department of Medicine West Virginia University Morgantown West Virginia USA

**Keywords:** anticoagulation, atrial fibrillation, CABG

## Abstract

**Background:**

The benefit of oral anticoagulation (OAC) in patients with new‐onset postoperative atrial fibrillation (POAF) after coronary bypass grafting remains uncertain. The 2023 American Heart Association (AHA) and the American College of Cardiology (ACC) guidelines recommend 60 days of anticoagulation for POAF, whereas management was previously left to the physician's discretion. The aim of this study is to identify the most effective anticoagulant regimens for reducing stroke rates in POAF patients after coronary artery bypass grafting (CABG).

**Methods:**

Using the Epic Cosmos database, we included all US patients who underwent CABG without concomitant aortic/mitral valve replacement (MVR) from 2017 to 2023. Primary outcomes include demographics of POAF/non‐POAF patients and complication rates among patients without anticoagulation, on direct oral anticoagulation (DOAC), or on warfarin.

**Results:**

Of the 147,546 patients studied, 7.6% (11,336) developed POAF. At 60 days, the use of OAC for POAF was associated with a decrease in all‐cause mortality without any difference in thromboembolism rates. At 12 months, there was no difference between all‐cause mortality and thromboembolism rates between the OAC and the no anticoagulation (NA) cohorts.

**Conclusions:**

The benefit of long‐term anticoagulation of POAF following CABG with warfarin or DOAC remains unclear, as there was no reduction in thromboembolic events with anticoagulation despite a reduction in all‐cause mortality at 60 days. No significant reduction in thromboembolic events or all‐cause mortality was exhibited at 1 year. Thus, the benefit of long‐term anticoagulation of POAF following CABG with warfarin or DOAC remains unclear.

**Abbreviations**
ACC: American College of Cardiology
ACEIs: angiotensin‐converting enzyme inhibitors
AHA: American Heart Association
ARBs: angiotensin II receptor blockers
AVR: aortic valve replacement
BMI: body mass index
CABG: coronary artery bypass graft
COPD: chronic obstructive pulmonary disease
DOAC: direct oral anticoagulation
EHR: electronic health record
ICD‐10: International Classification of Diseases, Tenth Revision
MVR: mitral valve replacement
NA: no anticoagulation
OAC: oral anticoagulation
POAF: postoperative atrial fibrillation
SD: standard deviation
TIA: transient ischemic attack

## 1 Introduction

Postoperative atrial fibrillation (POAF) is one of the most prevalent complications after a coronary artery bypass graft (CABG), characterized by new‐onset atrial fibrillation believed to be a direct consequence of the surgery. POAF can lead to serious complications, including stroke, increased mortality, and prolonged hospital stays. Critically, stroke rates can reach up to 18.2% in POAF patients, with a threefold increase in all‐cause mortality and a higher risk of stroke at 3 years (6.6% compared to 2.4%; hazard ratio 1.53) [1].

Anticoagulation is recommended for stroke prevention in atrial fibrillation patients with elevated embolization risk. Until December 2023, POAF anticoagulation management lacked definitive guidelines and was deferred to the physician's discretion. The latest American Heart Association (AHA) and American College of Cardiology (ACC) 2023 guidelines recommend oral anticoagulation (OAC) for at least 60 days in POAF patients [2]. OAC encompasses both warfarin and direct oral anticoagulation (DOAC). However, the efficacy of early anticoagulation on reducing thromboembolic events and all‐cause mortality remains unclear due to insufficient data and a lack of randomized controlled trials [3, 4].

This study aims to identify the most effective anticoagulant regimens for reducing stroke rates in POAF patients after CABG, excluding those with concurrent aortic or mitral valve replacement (MVR). We compared the demographics of POAF and non‐POAF patients and assessed complication rates among patients without anticoagulation, on DOAC, or on warfarin.

## 2 Methods

### 2.1 Ethical Approval

This study used de‐identified data from the Epic Cosmos database. Per institutional policy and federal regulations (45 CFR §46.104(d)(4)), this research was determined to be exempt from Institutional Review Board (IRB) review.

### 2.2 Data Sources

The Cosmos database aggregates over 295 million patient records across 198 USA healthcare organizations using Epic for electronic health records (EHRs). Cosmos database is a vendor‐facilitated (Epic, Verona, Wisconsin, USA) de‐identified database and is one of the largest nationwide health system databases currently available in the United States. The database currently comprises 1633 hospitals and 295 million patient records (Figure S1). It combines both inpatient and outpatient charts into a single record and aggregates data as patients move between health systems. We analyzed patients with a diagnosis or procedure of CABG or related billed procedures (List S1) from January 1, 2017 to December 31, 2023.

Patients were included if they developed atrial fibrillation/flutter (ICD 10 Diagnosis code I48*) within 1–31 days post‐CABG as an admitting, billed admitting, billed charge‐associated, billed final, or encounter diagnosis. Exclusions included concurrent CABG with other cardiac procedures (List S2) or a prior atrial fibrillation diagnosis before CABG. The dataset was reviewed for administration of warfarin or DOAC (dabigatran, rivaroxaban, apixaban, edoxaban, betrixaban) [4] during the same encounter, regardless of oral prescription status, due to incomplete data. Diagnosis was identified using ICD‐10 codes. We decided to include 1–31 days as a timeline to ensure inclusion of POAF.

**FIGURE 1:**
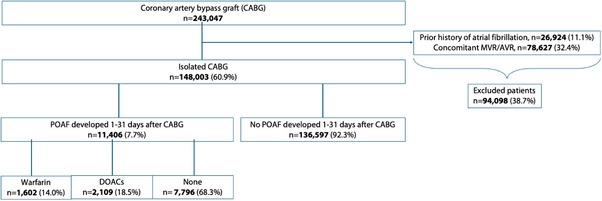
Flowchart of included and excluded patients. [Colour figure can be viewed at wileyonlinelibrary.com]

A total of 243,047 patients underwent CABG from January 1, 2017, to December 31, 2023. Among these, 78,627 patients underwent concomitant aortic valve replacement (AVR) or MVR and were excluded from the study. 26,924 patients had a prior history of atrial fibrillation and were also excluded. Our final cohort consisted of 148,003 patients who underwent isolated CABG (Figure 1). Patients who developed atrial fibrillation in the postoperative post‐operative period up to 31 days were identified. Subsequently, the cohort was divided into three groups depending upon the anticoagulation status: warfarin, DOAC, and no anticoagulation (NA). Primary outcomes were rates of all‐cause mortality, ischemic stroke, major bleeding (BARC 3–5), and hemorrhagic stroke at 60 days and 12 months.

### 2.3 Outcome Measures

The primary outcome of the study is to assess thromboembolic and bleeding complications in patients with and without anticoagulation at 60 days and 12 months. Secondary outcomes included all‐cause mortality. Diagnosis was identified using ICD‐10 codes (Table S1).

### 2.4 Statistics

For each demographic and outcome, the number of patients, 95% confidence interval, and standard error were collected. Descriptive statistics were presented as mean and standard deviation (SD) for continuous variables and as number and percentage for categorical variables. Pairwise chi‐square goodness‐of‐fit analyses were conducted on each demographic and outcome comparing patients on: warfarin vs. NA and DOAC vs. NA. A *p* value < 0.05 was considered significant.

Statistical analysis was performed in *R*, using the data table, dplyr, and rstatix packages. Plots and figures were produced using GraphPad Prism and *R* using tidyverse, pheatmap, knitr, gridBase, gridExtra, RColorBrewer, ggplot2, ggthemes, paletteer, ggsci, gplots, graphics, vcd, and corrplot.

## 3 Results

A total of 148,003 patients underwent isolated CABG between January 2017 and December 2023. 11,406 (7.7%) patients developed atrial fibrillation from post‐operative day 1 to day 31, out of which 32.7% were started on anticoagulation and 68.3% were not started on anticoagulation. In the anticoagulation group, 18.5% of patients were started on DOAC, while 14% of patients were started on warfarin.

**FIGURE 2:**
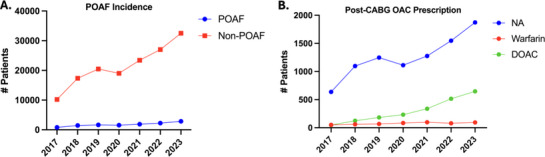
(A) POAF incidence over the years; (B) Use of OAC for CABG patients over the years. [Colour figure can be viewed at wileyonlinelibrary.com]

From January 1, 2017 to December 31, 2023, the proportion of CABG patients who develop POAF each year has remained relatively constant at around 7%–8% (Figure 2A). In contrast, the proportion of POAF patients that were prescribed an OAC has increased over time from 13.2% to 28.3%, with a preference of DOAC over warfarin (Figure 2B).

### 3.1 Patient Demographics

Patient characteristics are summarized in Table 1. Patients who developed POAF were older (mean age 71 ± 0.09 vs. 67 ± 0.76 years, *p* < 0.001). Additionally, patients with POAF had higher rates of comorbidities such as hypertension (86.4% vs. 85.4%, *p* = 0.0148), diabetes (43.2% vs. 47.9%, *p* < 0.001), heart failure (33.3% vs. 29.5%, *p* < 0.001), ischemic stroke (4.1% vs. 2.8%, *p* < 0.001), transient ischemic attack (TIA) (31.3% vs. 13.3%, *p* < 0.001), peripheral vascular disease (30.5% vs. 18.6%, *p* < 0.001), renal disease (34.5% vs. 27.4%, *p* < 0.001), and chronic obstructive pulmonary disease (COPD) (15.2% vs. 13.3%, *p* < 0.001). Notably, there was also a higher prevalence of sleep apnea among the POAF group (15.7% vs. 1.6%, *p* < 0.001).

**TABLE 1:** Comparison of demographics between patients who develop POAF and those who do not develop POAF following a CABG procedure.

Demographics of CABG patients	POAF (*n* = 11,406)	No POAF (*n* = 136,547)	*p* value POAF vs. no POAF
Age	71 (± 0.09)	67 (± 0.76)	< 0.001
BMI	31.3 (± 0.56)	31.6 (± 0.61)	0.717
Male sex	9190 (80.6)	102,803 (75.3)	< 0.001
History of hypertension	9851 (86.4)	116,611 (85.4)	0.015
History of vascular disease	11,394 (99.4)	136,021 (99.6)	0.435
History of diabetes	4933 (43.2)	65,351 (47.9)	< 0.001
History of heart failure	3799 (33.3)	40,288 (29.5)	< 0.001
History of ischemic stroke	464 (4.1)	3772 (2.8)	< 0.001
History of hemorrhagic stroke	23 (0.202)	140 (0.1)	0.016
History of TIA	3568 (31.3)	18,144 (13.3)	< 0.001
History of peripheral arterial embolism	119 (1)	911 (0.7)	0.341
History of peripheral vascular disease	3477 (30.5)	25,450 (18.6)	< 0.001
History of renal disease	3932 (34.5)	37,379 (27.4)	< 0.001
History of COPD	1729 (15.2)	18,109 (13.3)	< 0.001
History of liver disease	1974 (17.3)	5858 (4.3)	< 0.001
History of cancer	33 (0.289)	14,534 (10.6)	< 0.001
History of factor V Leiden	< 10	312 (0.2)	0.026
History of antiphospholipid syndrome	< 10	72 (0.1)	0.304
History of sleep apnea	1788 (15.7)	2175 (1.6)	< 0.001

*Note*: Number and proportions (%) or mean ± SD. POAF indicates postoperative atrial fibrillation. Significance is calculated from pairwise two‐tailed chi‐squared goodness‐of‐fit tests, except for age, which was calculated from a Welch two‐sample two‐tailed comparison of means *t*‐test.

Table 2 presents a comparison of demographics among POAF patients prescribed OAC (with warfarin or DOAC) vs. NA patients. Patients with a TIA and peripheral vascular disease were more likely to be prescribed DOAC (33.6% and 32.3%, respectively) compared to warfarin or NA patients (*p* < 0.001 and *p* = 0.043, respectively). Additionally, those on warfarin had a higher prevalence of renal disease compared to DOAC patients (40.4% vs. 37.5%, *p* = 0.074), although this was not statistically significant.

**TABLE 2:** Comparison of demographics between POAF patients who are prescribed OAC and those who are not.

Demographics of POAF	Warfarin (*n* = 1602)	DOAC (*n* = 2109)	NA (*n* = 7796)	Total (*n* = 11,406)	*p* value Warfarin vs. NA	*p* value DOAC vs. NA
Age	72 (± 0.38)	72 (± 0.19)	71 (± 0.1)	71 (± 0.09)	0.059	< 0.001
BMI	31.2 (± 0.29)	31 (± 0.14)	31.4 (± 0.74)	31.3 (± 0.56)	0.062	< 0.001
Male sex	1360 (84.9)	1747 (82.8)	6221 (79.8)	9190 (80.6)	< 0.001	< 0.001
History of hypertension	1469 (91.7)	1968 (93.3)	6580 (84.4)	9851 (86.4)	< 0.001	< 0.001
History of vascular disease	1602 (100)	2109 (100)	7788 (99.9)	11394 (99.4)	0.103	0.141
History of diabetes	636 (39.7)	910 (43.1)	3391 (43.5)	4933 (43.2)	0.997	0.775
History of heart failure	598 (37.3)	758 (35.9)	2526 (32.4)	3799 (33.3)	0.001	< 0.001
History of ischemic stroke	67 (4.2)	99 (4.7)	304 (3.9)	464 (4.1)	0.287	0.101
History of hemorrhagic stroke	< 10	< 10	19 (0.24)	23 (0.202)	−	0.023
History of TIA	182 (33.6)	691 (33.6)	2401 (30.8)	3568 (31.3)	0.014	0.084
History of peripheral arterial embolism	< 10	24 (1.1)	77 (1.0)	119 (1.0)	−	0.542
History of peripheral vascular disease	511 (31.9)	681 (32.3)	2339 (0.992)	3477 (30.5)	0.193	0.043
History of renal disease	647 (40.4)	791 (37.5)	2604 (33.4)	3932 (34.5)	< 0.001	< 0.001
History of COPD	210 (13.1)	379 (18)	1146 (14.7)	1729 (15.2)	0.952	< 0.001
History of liver disease	139 (8.7)	171 (8.1)	663 (8.5)	1974 (17.3)	0.397	0.561
History of cancer	322 (20.1)	393 (18.6)	1310 (16.8)	33 (0.289)	0.001	0.048
History of factor V Leiden	< 10	< 10	16 (0.205)	< 10	−	0.037
History of sleep apnea	263 (16.4)	343 (16.3)	1208 (15.5)	1788 (15.7)	0.183	0.39

*Note*: Number and proportions (%) or mean ± SD. POAF indicates postoperative atrial fibrillation. DOAC indicates direct oral anticoagulation. Significance is calculated from pairwise two‐tailed chi‐squared goodness‐of‐fit tests, except for age, which was calculated from a Welch two‐sample two‐tailed comparison of means *t*‐test.

### 3.2 Postoperative POAF Anticoagulation and Outcomes

Outcomes were analyzed at 60 days and 2–12 months to account for perioperative complications (Table 3). At 60 days, the warfarin and DOAC cohorts had reduced all‐cause mortality of 2.2% and 2.3% compared to 4.6% in the NA group (*p* < 0.001 for both cohorts). However, there was no difference in all‐cause mortality at 12 months. Rates of thromboembolism (e.g., ischemic stroke, peripheral arterial embolism, and pulmonary embolism or deep vein thrombosis) at both 60 days and 12 months were not significantly reduced in patients on OAC. Furthermore, no difference between rates of all‐cause mortality exists between warfarin and DOAC cohorts in comparison to the NA group at 12 months. Warfarin was associated with higher rates of major bleeding compared to DOAC in the first year (*p* = 0.0046). There was also a significantly higher rate of hemorrhagic stroke in the DOAC group compared to the NA group (*p* = 0.0256).

**TABLE 3:** Comparison of POAF complication rates between patients on different types of anticoagulation.

Outcomes 1–60 days	Warfarin (*n* = 1602)	DOAC (*n* = 2109)	NA (*n* = 7796)	Total (*n* = 11,406)	*p* value Warfarin vs. NA	*p* value DOAC vs. NA
Death	35 (2.2)	49 (2.3)	356 (4.6)	440 (3.9)	< 0.001	< 0.001
Major bleeding	155 (9.7)	204 (9.7)	704 (9)	1052 (9.2)	0.449	0.384
Ischemic stroke	83 (5.2)	103 (4.9)	346 (4.4)	527 (4.6)	0.209	0.376
Hemorrhagic stroke	< 10	< 10	27 (0.3)	37 (0.3)	0.0358	0.0139
Peripheral arterial embolism	20 (1.3)	13 (0.623)	51 (0.7)	83 (0.7)	0.0185	0.989
Pulmonary embolism/ deep vein thrombosis	57 (3.6)	53 (2.5)	246 (3.1)	354 (3.1)	0.424	0.136
Heart failure	525 (32.7)	615 (29.2)	2036 (26.1)	3134 (27.5)	< 0.001	0.0057
TIA	133 (8.3)	171 (8.1)	576 (7.4)	869 (7.61)	0.241	0.302

Outcomes 2–12 months	Warfarin (*n* = 1602)	DOAC (*n* = 2109)	NA (*n* = 7796)	Total (*n* = 11,406)	*p* value Warfarin vs. NA	*p* value DOAC vs. NA
Death	40 (2)	49 (2.3)	171 (2.2)	258 (2.3)	0.503	0.745
Major bleeding	203 (12.7)	204 (9.7)	746 (9.6)	1142 (10.0)	< 0.001	0.913
Ischemic stroke	85 (5.3)	104 (4.9)	341 (4.4)	521 (4.6)	0.106	0.297
Hemorrhagic stroke	< 10	< 10	23 (0.296)	35 (0.3)	0.058	0.026
Peripheral arterial embolism	20 (1.3)	17 (0.814)	61 (0.784)	97 (0.9)	0.089	1.00
Pulmonary embolism/ deep vein thrombosis	53 (3.3)	42 (2)	196 (2.5)	290 (2.5)	0.0825	0.207
Heart failure	569 (35.5)	672 (31.8)	2,211 (28.4)	3409 (29.9)	< 0.001	0.002
TIA	170 (10.6)	257 (12.2)	803 (10.3)	1220 (10.7)	0.749	0.016

*Note*: Number and proportions (%). DOAC indicates direct oral anticoagulation. Significance calculated from Pearson's pairwise two‐tailed chi‐squared goodness‐of‐fit tests.

### 3.3 Medications on Discharge

Table 4 compares discharge medications prescribed to POAF patients on warfarin, DOAC, or NA following CABG. Cardio‐selective beta‐blockers were the most prescribed medication across all groups, with 45.5% of warfarin patients, 53.1% of DOAC patients, and 39.1% of NA patients receiving them (*p* < 0.001 for both comparisons). Antiplatelet use was also significantly different between warfarin and DOAC patients (27.9% vs. 27.9%, *p* < 0.001), with both groups having lower usage than the NA group. Similarly, class III antiarrhythmics, including amiodarone, were prescribed significantly more often in DOAC and warfarin groups (61.5% and 53.4%, respectively) compared to the NA group (32.3%, *p* < 0.001). There was no significant difference in the use of angiotensin‐converting enzyme (ACE) inhibitors/angiotensin II receptor blockers (ARBs), aldosterone antagonists, or digoxin across all groups.

**TABLE 4:** Comparison of discharge medications prescribed to POAF patients on warfarin, DOAC, or NA following CABG.

Discharge medications	Warfarin (*n* = 1602)	DOAC (*n* = 2109)	NA (n = 7796)	Total POAF (*n* = 11,406)	Total non‐POAF (*n* = 136,597)	*p* value Warfarin vs. DOAC	*p* value DOAC vs NA
ACEIs/ARBs	165 (10.3)	255 (12.1)	702 (9)	1118 (9.8)	15,413 (11.2)	0.088	< 0.001
Beta blockers cardioselective	729 (45.5)	1120 (53.1)	3048 (39.1)	4848 (42.5)	60,274 (43.8)	< 0.001	< 0.001
Beta blockers non‐cardioselective	< 10	< 10	11 (0.14)	19 (0.17)	96 (0.07)	−	0.084
Aldosterone antagonists	42 (2.6)	53 (2.5)	133 (1.7)	228 (2)	2615 (1.9)	0.836	0.015
Antiplatelets	368 (23)	588 (27.9)	2237 (28.7)	3171 (27.8)	48,027 (34.9)	< 0.001	0.463
Vitamin K antagonists	995 (62.1)	25 (1.2)	< 10	992 (8.7)	1273 (0.93)	−	< 0.001
Lipid‐lowering agents	397 (24.8)	654 (31)	1894 (24.3)	2920 (25.6)	42,935 (31.2)	< 0.001	< 0.001
Digoxin	30 (1.9)	59 (2.8)	19 (0.24)	105 (0.92)	253 (0.18)	0.068	< 0.001
Calcium channel blockers	111 (6.9)	221 (10.5)	491 (6.3)	821 (7.2)	9220 (6.7)	< 0.001	< 0.001
All antiarrhythmics	1065 (66.5)	157 (7.45)	3921 (50.3)	6579 (56.8)	66,604 (48.4)	< 0.001	< 0.001
Class I antiarrhythmics	< 10	< 10	< 10	< 10	50 (0.04)	−	−
Class III antiarrhythmics	855 (53.4)	1297 (61.5)	2518 (32.3)	4608 (40.4)	17,890 (13)	< 0.001	< 0.001
Amiodarone	852 (53.2)	1295 (61.4)	2510 (32.2)	4597 (40.3)	17,890 (13)	< 0.001	< 0.001

*Note*: Number and proportions (%). DOAC indicates direct oral anticoagulation. Significance calculated from Pearson's chi‐squared goodness‐of‐fit tests.

## 4 Discussion

The main findings of this study include as follows: (1) OAC was initiated in 32.7% of post‐CABG POAF patients, (2) anticoagulation therapy was not associated with a reduction in risk for thromboembolic events, but with a reduced risk of all‐cause mortality at 60 days.

The proportion of patients on OAC in this study was higher than reported in other studies, such as 23.7% [3] and up to 37% in a systematic review and meta‐analysis [5]. The prevalence of POAF following CABG was 7.7%, which is lower than some studies [3] but falls within previously reported ranges [5, 6]. In our current study, there was a rise in anticoagulation prescription for POAF patients from 2017 to 2023, which corresponds to trends previously reported in literature [7]. However, our findings suggest limited benefits of long‐term OAC use in this population. While OAC use was associated with a decrease in all‐cause mortality at 60 days, there was no statistically significant difference in thromboembolism rates. This aligns with the 2023 AHA/ACC guidelines to prescribe anticoagulants to POAF patients for 60 days following a CABG procedure.

Neither DOAC nor warfarin significantly reduced the risk of ischemic stroke. The lack of significant differences in rates of thromboembolism between 2 and 12 months post‐CABG calls into question the utility of long‐term anticoagulation. While warfarin and DOAC are expected to reduce complications associated with blood coagulability, similar findings have been reported in other studies, including an analysis using the SWEDEHEART Registry [3], which also found no significant reduction in mortality or thromboembolic events with OAC use. Another meta‐analysis [6] echoed these findings, demonstrating no significant differences in thromboembolism or mortality between POAF patients treated with OAC and those without anticoagulation. The NOAH‐AFNET 6 trial similarly concluded that early anticoagulation did not reduce death, stroke, or systemic embolism compared with placebo [7].

In our current study, in terms of safety, the risk of major bleeding with OAC was slightly elevated but not statistically significant in the first 60 days. However, this risk increased significantly during the 2–12‐month period. While Almassi et al. found no association between OAC and bleeding complications [8], other studies reported an increased risk of major bleeding with early anticoagulation [3, 6].

When comparing the demographics of patients with and without POAF, known risk factors for atrial fibrillation, such as sleep apnea, heart failure, and chronic kidney disease, were more prevalent in the POAF group, as expected. Filardo et al. found that men were more likely to develop POAF than women, [9] as we found, but women had higher rates of stroke and mortality [10].

This study highlights the value of the Epic Cosmos dataset in examining nationwide, multicenter‐based treatment patterns. While similar studies have been performed in Europe [3], this is the first database study on CABG patients in the United States of America based on AHA and ACC guidelines.

The Cosmos database offers significant advantages for health research due to its vast scale across diverse healthcare settings, allowing for extensive and generalizable research on various medical conditions and treatments. It provides real‐world clinical data, enabling large‐scale studies of everyday healthcare practices, and tracking of longitudinal outcomes. While Epic Cosmos has been used to compare treatments for atrial fibrillation [5], no studies have been published on the treatment of POAF.

However, the database also has limitations. As with any EHR‐based dataset, it is susceptible to missing data, variable data quality, and inconsistencies due to differences in how information is recorded across institutions. The diagnosis of atrial fibrillation was determined using the ICD‐10 code, which precluded the assessment of atrial fibrillation episode duration. This reliance on coding may also contribute to a potentially lower overall incidence of atrial fibrillation observed in the study, likely due to inherent coding errors and documentation variability. Mortality is likely undercounted if it is not consistently reported in a patient's EHR record, which may affect overall the mortality outcome and average age.

Regarding medication data, medication adherence cannot be reliably measured beyond prescription records since each institution receives external medication information differently, and the data is subject to the limitations of coding accuracy and completeness. A specific limitation pertinent to this study was the inability to establish and include pre‐operative medication data, such as amiodarone, due to database constraints. While patients with a prior atrial fibrillation diagnosis, a condition that would inherently obviate the need for pre‐operative amiodarone, were excluded from the study, it is recognized that post‐operative prophylactic amiodarone administration is a common practice that could potentially contribute to a decreased incidence of atrial fibrillation. However, temporal data precisely linking the occurrence of atrial fibrillation with the initiation of amiodarone therapy was unavailable within the dataset. The database is dynamic, with continuously updating data points, which can lead to challenges in maintaining consistency in longitudinal studies. Furthermore, key clinical variables, like CHA_2_DS_2_‐VASc scores or left ventricular ejection fractions, were insufficiently populated relative to the sample size, limiting the depth of certain analyses. Aggregated summary statistics data means that adjusting for covariates in this analysis was not possible. Despite these challenges, the Epic Cosmos database remains a powerful tool for large‐scale health research, particularly when these limitations are carefully managed.

Future research could explore the impact of medication dosage, duration, prior regimens, and longitudinal prescription trends. Expanding this analysis across other biobanks could refine clinical risk scores for POAF and guide future randomized controlled trials to establish causality.

## 5 Conclusion

The benefit of long‐term anticoagulation with warfarin or DOAC remains unclear in patients with post‐operative atrial fibrillation after isolated CABG. In our study, there was no reduction in thromboembolic events with anticoagulation. However, there was a reduction in all‐cause mortality at 60 days that was no longer present at 1 year. Future studies and randomized controlled trials are needed to optimize the duration of anticoagulation for POAF.

## Conflicts of Interest

The authors declare no conflicts of interest.

## Supporting information




**Supporting List 1**: List of CABG procedures.
**Supporting List 2**: List of MVR/AVR procedures.
**Supporting Table 1**: List of codes.
**Supporting Figure 1**: Geographical representation of Cosmos cohort that received an isolated CABG in United States 2017–2023.

## Data Availability

Anonymized data and materials are available in the Epic Cosmos database.
