# Supplementary material for: Trends and Outcomes of Anticoagulation for Post‐Operative Atrial Fibrillation After Coronary Artery Bypass Graft
Source: Pacing Clin Electrophysiol. 2025 Oct 23;48(12):1444–50. doi: 10.1111/pace.70069 (PMC12671470; doi:10.1111/pace.70069)

**SUPPLEMENTAL**

Supplemental List 1: List of CABG procedures

- ABD-CORON ARTERY BYPASS
- CABG W/ARTERIAL GRAFT FOUR/>ARTERIAL GRAFTS 33536
- CORONARY ARTERY BYP W/VEIN &ARTERY GRAFT 6 VEIN 33523
- CORONARY ARTERY BYPASS 4 CORONARY VENOUS GRAFTS 33513
- CORONARY ARTERY BYP W/VEIN & ARTERY GRAFT 1 VEIN 33517
- HARVEST UPPER EXTREMITY ARTERY 1 SEGMENT CABG 35600
- HARVEST UXTR VEIN 1 SGM LOWER EXTREMITY/CABG PX 35500
- CABG W/ARTERIAL GRAFT SINGLE ARTERIAL GRAFT 33533
- CABG W/ARTERIAL GRAFT TWO ARTERIAL GRAFTS 33534
- LIMA GRAFT USED IN 1ST ISOLATED CABG PXD 4110F
- CORONARY ARTERY BYPASS 6/+ CORONARY VENOUS GRAFT 33516
- RPR ANOM CORONARY ARTERY PULM ART ORIGIN GRAFT 33503
- STK/CVA CABG G8573
- CORONARY ARTERY BYP W/VEIN & ARTERY GRAFT 4 VEIN 33521
- CABG W/ARTERIAL GRAFT THREE ARTERIAL GRAFTS 33535
- CORONARY ARTERY BYPASS 1 CORONARY VENOUS GRAFT 33510
- CORONARY ARTERY BYPASS 2 CORONARY VENOUS GRAFTS 33511
- CORONARY ARTERY BYP W/VEIN & ARTERY GRAFT 2 VEIN 33518
- CORONARY ARTERY BYPASS 3 CORONARY VENOUS GRAFTS 33512
- NDSC SURG W/VIDEO-ASSISTED HARVEST VEIN CABG 33508
- CORONARY ARTERY BYP W/VEIN & ARTERY GRAFT 5 VEIN 33522
- Bypass Coronary Artery, One Site from Cor Art, Open Approach
- CORONARY ARTERY BYP W/VEIN & ARTERY GRAFT 3 VEIN 33519
- CORONARY ARTERY BYPASS 5 CORONARY VENOUS GRAFTS 33514
- Bypass Coronary Artery, One Site from Abd Art, Open Approach

Supplemental List 2: List of MVR/AVR procedures

- Replacement of Mitral Valve with Autol Sub, Open Approach
- Replacement of Mitral Valve with Synth Sub, Open Approach
- Replacement of Mitral Valve with Nonaut Sub, Open Approach
- Replacement of Mitral Valve with Zooplastic, Open Approach
- Repair Mitral Valve, Open Approach
- Replacement of Aortic Valve with Autol Sub, Open Approach
- Replacement of Aortic Valve with Synth Sub, Open Approach
- Replacement of Aortic Valve with Nonaut Sub, Open Approach
- Replacement of Aortic Valve with Zooplastic, Open Approach
- REPLACE AORTIC VALVE OPEN TRANSTHORACIC APPROACH 0318T
- Repair Aortic Valve, Open Approach
- Release Aortic Valve, Open Approach
- REPLACE AORTIC VALVE OPEN TRANSAORTIC APPROACH 33365

Supplemental Table 1: List of codes

| Hypertension | I10.*, I11.*, I12.*, I13.*, I15.* |
| --- | --- |
| Vascular disease | I21, I22, I25, I70-73 |
| Diabetes | E10.*-E13.* |
| Heart failure | I50.* |
| Ischemic stroke | I63.* |
| Hemorrhagic stroke | I61.* |
| Transient ischemic attack | I66.*, I65.*, G45.* |
| Peripheral arterial embolism | I74.* |
| Peripheral vascular disease | I70.* -I74.*, I77.* |
| Renal disease | N17-19 |
| COPD | J44.* |
| Liver disease | K70-77  Alcohol - F10, G312, G621, G721, K292, K70, K860, O354 |
| Cancer | Z85.* |
| Antiphospholipid syndrome | D68.61 |
| Factor V Leiden | D68.51 |
| sleep apnea | G47.33 |
| Major bleeding | - Intracranial bleeding - I60, I61, I62, I690, I691, I692 - GI Bleeding - I850, I983, K226, K250, K252, K254, K256, K260, K262, K264, K266, K270, K272, K274, K276, K280, K284, K290, K625, K661, K920, K921, K922, K25, K26, K27, K28, I850, I983, K221,K226 - Hemopericardium - I230, I312 - Hemothorax - J942 - Urogenital bleeding - N02, R319, N95, N939, N501A - Other - H431, R04, R58, D629, T810, DR029, D50 |
| Pulmonary embolism/  deep vein thrombosis | I26, I82.4* |

Supplemental Figure 1: Geographical representation of Cosmos cohort that received an isolated CABG in United States 2017-2023


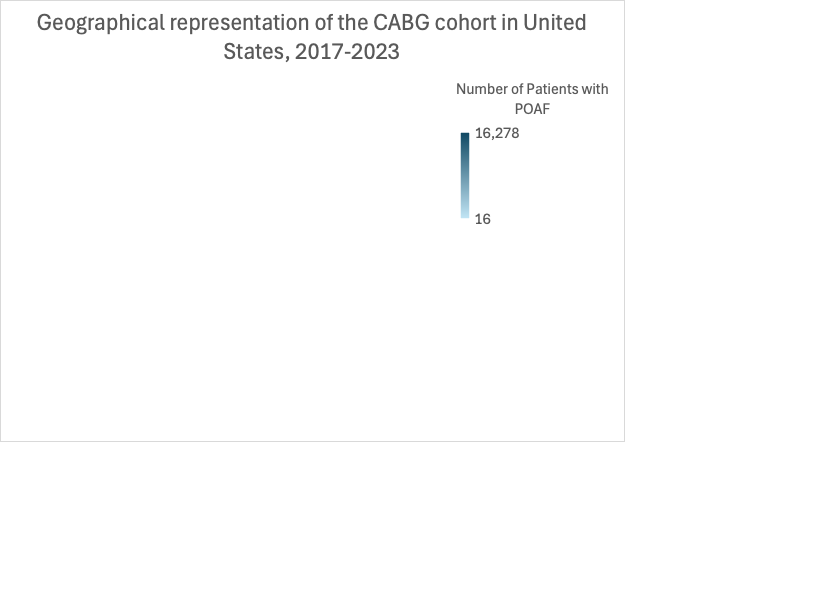

Supplement: Supplementary file 1 — Supporting List 1: List of CABG procedures. Supporting List 2: List of MVR/AVR procedures. Supporting Table 1: List of codes. Supporting Figure 1: Geographical representation of Cosmos cohort that received an isolated CABG in United States 2017–2023. [file PACE-48-1444-s001.docx]
